# Micro-CT Assessment of Heat and Vibration Effect on Sealant Penetration in Different Fissure Types

**DOI:** 10.3290/j.ohpd.c_1816

**Published:** 2025-01-23

**Authors:** Krasimir Mitkov Hristov, Liliya Marinova Angelova, Nedana Emilova Georgieva, Ralitsa Todorova Bogovska-Gigova

**Affiliations:** a Krasimir Mitkov Hristov Associate Professor, Department of Pediatric Dentistry, Faculty of Dental Medicine, Medical University of Sofia, Sofia, Bulgaria. Study design and implementation, performing data extraction and synthesising the results, writing the manuscript, and final approval of the version to be submitted.; b Liliya Marinova Angelova Assistant Professor, Department of Dental Public Health, Faculty of Dental Medicine, Medical University of Sofia, Sofia, Bulgaria. Formal analysis, visualisation, manuscript review and editing.; c Nedana Emilova Georgieva Assistant Professor, Department of Pediatric Dentistry, Faculty of Dental Medicine, Medical University of Sofia, Sofia, Bulgaria. Data extraction.; d Ralitsa Todorova Bogovska-Gigova Assistant Professor, Department of Pediatric Dentistry, Faculty of Dental Medicine, Medical University of Sofia, Sofia, Bulgaria. Performing data extraction and synthesising the results, analysing and interpreting data, and writing the manuscript.

**Keywords:** caries prevention, sealant penetration, pits and fissures, sonic vibration, heat

## Abstract

**Purpose:**

To make micro-CT comparison and evaluation of sealant penetration depth in different types of fissures after heating of the material or application of vibrations.

**Materials and Methods:**

One hundred sound third molars have been sealed as follows: group 1 (n = 20), light-cured resin sealant at room temperature, group 2 (n = 20), light-cured resin sealant, preheated to 41.0°C, group 3 (n = 20), light-cured resin sealant, preheated to 51.0°C, group 4 (n = 20), resin sealant with application of vibrations before light-curing at room temperature, group 5 (n = 20), resin-modified glass-ionomer cement. The samples were analysed using micro-computed tomography (micro-CT). The profile of each fissure was classified, and the penetration depth of the sealant into the fissure and the fissure depth were measured. The ratio of filled area and total depth of the fissure was calculated in percentages.

**Results:**

Pre-heating of the sealants and the usage of a vibrating tool improved the penetration depth compared to the application of the material at room temperature. U- and V-type fissures exhibited better penetration capability than others. For IK-type fissures, the best penetration was observed with resin sealant heated at 51.0°C. I-shaped fissures exhibited lower penetration rates despite the heating process. Glass-ionomer cement showed the least depth penetration.

**Conclusion:**

Pre-heating of the resin sealant or application of vibrations improve statistically significantly penetration in the different fissure types.

Dental caries is a multifactorial disease caused by a change in the composition of the biofilm, leading to an imbalance between the processes of de- and remineralisation.^21^ Occlusal caries lesions account for approximately 50 to 90% of all lesions found in permanent teeth, despite the fact that the pits and fissures in permanent molars occupy only 22.30% and 6.52% of the entire dental crown.^10,13,28^ The intricate occlusal morphology characterised by extensively branched sulci and deep pits and fissures creates an optimal environment for the accumulation of plaque and food particles, while simultaneously being difficult to reach through mechanical cleaning methods.^21,25^

Sealants offer a mechanical barrier to obstruct plaque accumulation on the occlusal surface, thus hindering or arresting the progression of dental caries.^10,26^ The main advantages of these materials are that they are cost-effective, prevent the need for more expensive and invasive restorative treatment, and reduce the risk of caries up to nine times.^7,9^ Dental professionals must recognise the widespread nature of occlusal carious lesions and consider sealants as a standard preventive measure.^1^

The occlusal fissure pattern is classified based on its morphology into five types:^22^ (1) V-type – wide at the top and gradually narrowing towards the bottom; (2) U-type – almost the same width from top to bottom; (3) I-type – extremely narrow slit; (4) IK-type – extremely narrow slit with a larger space at the bottom; (5) Other types that are not described in the types just mentioned. While some configurations are broad and shallow, facilitating self-cleansing, others are narrow and deep, making them more prone to develop caries lesions.^1^ However, due to morphological variation in different areas of the occlusal surface, it is not always possible to categorise a tooth as having a particular type of fissure.^1^ The detailed microstructure of these fissures is pivotal in assessing their vulnerability to lesion development and progression rate.^22^

Resin-based materials or glass-ionomer cement are the most common choices for fissure sealing.^27^ A sealant’s ability to penetrate the pits and fissures is governed by factors such as the shape and morphology of the fissures and the properties of the materials used.^6,11^ Retention of sealants is an integral requirement for their success.^5^ Sealant retention depends on a significant degree of its penetration rate.^15,23^ Incomplete retention of materials may be associated with a risk of carious lesion formation or progression.^3,5,20^ The sealant’s ability to seal the fissure depends on the depth of penetration, the morphology of the fissures, and the properties of the materials used.^24^

There is no data-driven support for any particular method of sealant placement. Care should be taken to avoid incorporating tiny air bubbles within the sealant, which will result in voids.^7^ The sealant material can be applied to the tooth in various methods. Many sealant manufacturers have their dispensers or applicators for sealant placement. When using a dispenser, the dentist should allow the sealant to flow ahead into the fissure as the dispenser is advanced from one end of the tooth to the other. This minimises the entrapment of air bubbles.^2^

Resin composite heating has been proposed to increase the physical properties and lower material viscosity.^4,16,18^ Current literature is sparse regarding studies that assess how changing sealant viscosity affects penetration rates. Therefore, the aim of this study was to evaluate sealant penetration depths through micro-CT analysis, considering the fissure type and comparing three application approaches: traditional, heating of the material, and application of vibrations.

## MATERIALS AND METHODS

One hundred permanent human third molars with occlusal morphology resembling the anatomy of a first permanent molar were included in the study. The teeth were extracted due to orthodontic reasons. After extraction, tooth crowns were swabbed with hydrogen peroxide and stored in a 10% formalin solution.^17^

Criteria for inclusion of the third molars in the study:

Intact occlusal surface without visible fractures, dysplastic defects, and crown cracks;
Absence of carious lesions, fillings, or sealants;
Macroscopic anatomy, similar to a first permanent molar.

Prior to silanisation, the occlusal surface and fissure and pit system were cleaned with pressurised sodium bicarbonate suspension (PROPHYflex 3, Kavo, Biberach, Germany) and washed with an air/water syringe to remove any residual debris or contamination that would interfere with the full penetration of the sealant. The samples were then observed with an operating microscope (Semorr 3000E, Semorr Medical Tech, Jiangsu, China) to ensure that the fissures were contamination free and without occlusal carious lesions. The occlusal surface was etched for 30 s with 37% orthophosphoric acid and washed with water for 30 s. The teeth thus prepared were then randomly divided into five groups, ensuring an unbiased approach to the study:

First group (20 permanent third molars, control group) – application with a micro-cannula of resin sealant Grandio Seal (VOCO, Cuxhaven, Germany) at room temperature (21.5°C with air-conditioner) in the entrance of the fissures and pits, waiting 10 s, removing the excess with a dry cotton pellet, and curing for 20 s with a light-curing lamp.
Second group (20 permanent third molars) – application with a micro-cannula of Grandio Seal resin sealant at the pits and fissures entrance after heating it to 41.0°C using a resin composite heater (AzDent Dental Composite Resin Heater, Henan Baistra Industries Corp, ZHENGZHOU, China), waiting 10 s, removing excess with a dry cotton pellet and curing for 20 s.
Third group (20 permanent third molars) – application of Grandio Seal resin sealant using a micro-cannula at the pits and fissures entrance after heating it to 51.0°C using a composite heater, waiting for 10 s, removing the excess with a dry cotton pellet and curing for 20 s.
Fourth group (20 permanent third molars) – application of Grandio Seal resin sealant with a micro-cannula at the pits and fissures entrance at room temperature, followed by 140 Hz vibrations applied along the fissure system using a vibrating instrument with a conical tip (Compothixo, Kavo Kerr, California, USA) for 10 s, removal of excess with a dry cotton pellet and curing for 20 s.
Fifth group (20 permanent molars, control group) – application of resin-modified glass-ionomer cement (GIC) Fuji Triage (GC Corp, Tokyo, Japan) at the pits and fissures entrance, prepared according to the manufacturer’s requirements, spreading carefully with a brush, waiting for 10 s and curing for 20 s.

**Fig 1:**
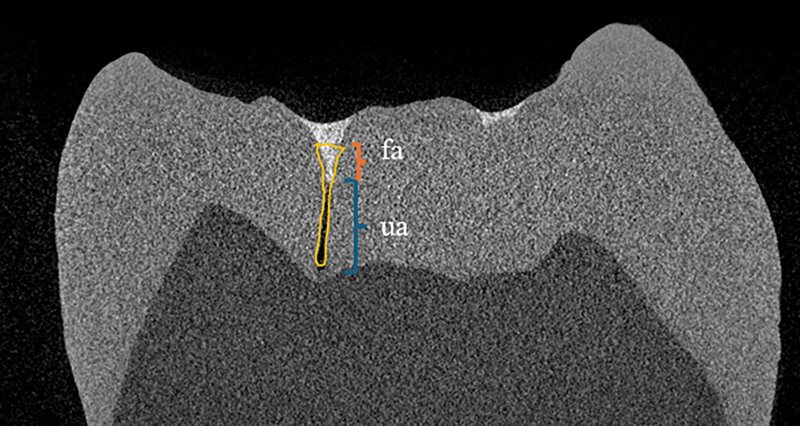
Penetration depth measurement (fa – filled area, ua – unfilled area).

The examined samples were fixed with silicone impression material and scanned with a desktop X-ray microtomograph SkyScan 1272 (Bruker, Billerica, MA, United States), X-ray tube voltage 100 kV, and current magnitude 100 µA with a 0.55 mm copper filter. The radiation was conical in shape, and the size of a single voxel was 12 µm. The crowns projected their entire length into the detector field at this resolution. On the obtained two-dimensional images, the profile of the fissure, the penetration depth of the sealant and the depth of the unfilled part of the fissure were evaluated (Fig 1). To standardise the measurement, a horizontal reference line was drawn using the micro-CT software and the entrance of the fissure was defined when the occlusal width was 0.5 mm.^14^ The filled area (fa) was measured from the entrance of the fissure to the deepest point where the sealant reached. The unfilled area (ua) was measured from the deepest point, where the sealant was found, to the bottom of the fissure. The filled and unfilled area (fa + ua) accounted for the depth of the fissure.

Penetration depth was calculated using the following formula (Fig 1):

penetration (%) =

fa

fa + ua

∙

100

### Statistical Analysis

The data were analysed using a non-parametric test (Mann–Whitney U Test, Chi-squared test) because assumption of normality could not be met (Shapiro–Wilk test, p < 0.05). The significance level was set at p = 0.05. Statistical analysis was conducted with a statistics computer software SPSS v.19.0 (SPSS, Chicago, IL, USA).

## RESULTS

**Fig 2:**
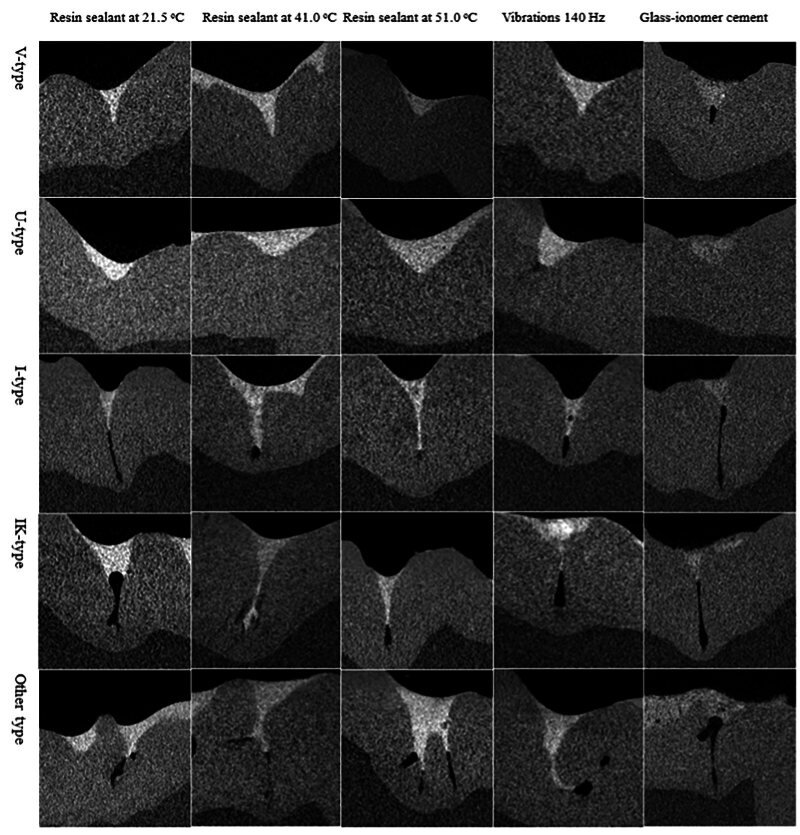
Sealant penetration in fissures with various profiles. The application of mechanical or thermal energy lowers the viscosity and allows the sealant to infiltrate the pits and fissures system more effectively.

Figure 2 presents the difference in the penetration rate of Grandio Seal resin composite sealant and Fuji Triage GIC into different types of fissures after exposure to heat and vibration.

**Fig 3:**
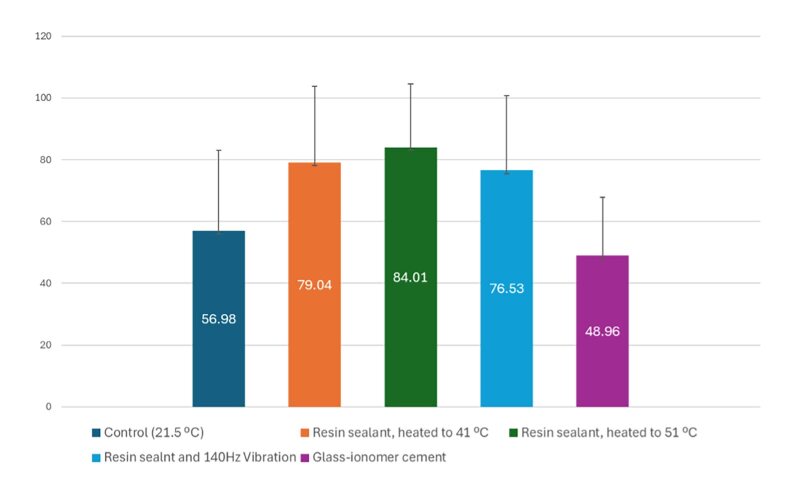
Comparison of sealant penetration rates between different groups. Different lowercase letters indicate significant differences (p < 0.05; Mann–Whitney U test).

From the general comparison of the degree of sealant penetration in the depth of the fissure, statistically significant differences were found among all the studied groups (Fig 3). The penetration rate of sealants varied on average between 48% and 84%, with the most significant penetration shown by heated materials – 79% penetration when heated at 41.0°C and an even greater depth at 51–84%. The lowest penetration in depth was recorded when applying GIC as a sealant and it filled on average less than half of the depth of the fissure. The control group at 21.5°C showed penetration close to that of GIC.

When comparing the penetration depth in V-shaped fissures, the sealants showed statistically significantly greater penetration after heating or applying vibrations – from 86% to 89%. GIC and the resin sealant at room temperature had the worst penetration into the fissure system – 58% and 68%, respectively.

The trend observed in V-type fissures was preserved also in U-shaped fissures. The sealant was heated at 41.0°C and 51.0°C, and the application of vibrations led to almost complete penetration of the sealant in the fissure depth – over 97%, without statistically significant differences between the three studied groups. A statistically significantlylower degree of penetration was observed with GIC in U-shaped fissures – more than half of the depth of the fissure remains unfilled. At room temperature, the resin sealant filled more than 80% of the fissure depth.

I-shaped fissures have a lower penetration rate and most of the fissure remained unfilled when the resin sealant was at 21.5°C or GIC was used. The penetration improved when heat or vibrations were applied. No statistically significant difference was observed when vibration method of application was used or pre-heating at 41°C. The sealant reaches up to and a little over 60% of the depth of the fissure. The highest penetration was found in the group where a sealant heated to 51°C was used.

IK-type fissures were filled from 27 to 66%, and the most suitable material was the resin sealant, regardless of the method of application – with or without heating, or with application of vibration. No statistically significant differences were found when comparing the first (room temperature) to the next three groups (heating at 41°C, 51°C, and vibrations at room temperature) when resin sealant was used.

When the fissure profile is classified as another type, a statistically significant variation in the penetration depth of the investigated materials can be seen. The greatest depth of penetration was recorded when the sealant was heated to 51.0°C and the lowest – when GIC was used.

## DISCUSSION

**Fig 4:**
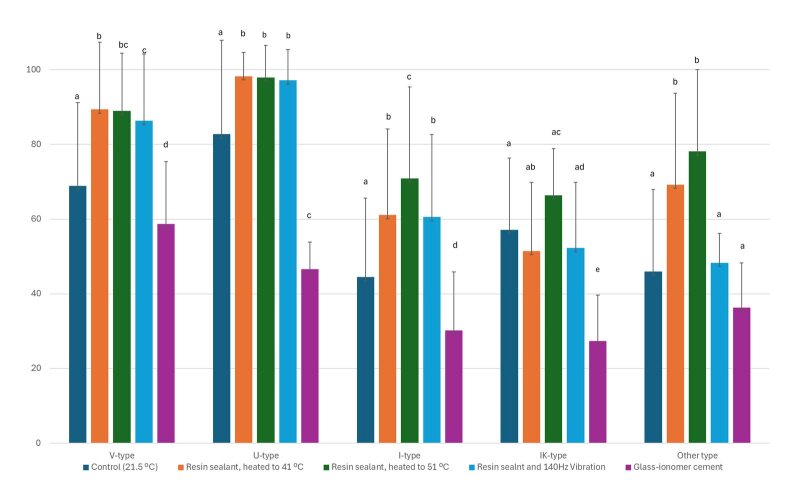
Comparison of sealant penetration rate in different fissure profiles. Different lowercase letters indicate significant difference (P < 0.05; Mann–Whitney U test).

The depth, complexity, and fissure type can affect sealant penetration.^23^ Our study found that, regardless of the fissure profile, GIC showed the lowest degree of penetration, varying from 27% to 58% across fissure profiles (Fig 3). A group of authors studied and compared the penetration ability of three commercially available pit and fissure sealants and found that, of all materials, penetration was most significant in the U-type fissure pattern and averaged 93.89%.^10^ In second place is the V-type fissure pattern with 78.62%. IK-type and I-type fissures showed 74.34 and 65.91% penetration, respectively.^10^ According to them, the penetration depth of GC Fuji VII glass-ionomer sealant was the best.^10^ Our study showed the opposite results, and the Fuji Triage glass-ionomer sealant showed penetration in only half of the fissure (Fig 4). According to a study assessing the sealing ability of sealants in different fissure morphologies, the penetrability of moisture-tolerant sealant in V-type fissures is 89.8%, and glass-ionomer sealant is 79.5%, whereas in I-type fissures, the penetrations are 81.6% and 65.1%, respectively.^24^

Compothixo vibrating tool technology (Kavo Kerr, CA, USA) improves the thixotropic properties of composites by changing only their viscosity without affecting the chemical and mechanical properties of the material. In our study, we found that the fissure depth penetration of the sealant by using this type of vibrating tool statistically significantly increased compared to the control groups in most fissure types (Fig 4). Exceptions were the fissures classified as another type, where no statistical difference was found, and those with an IK-type profile, where the penetration in the control group at room temperature was statistically significant. Vibrating assistance in sealant application is recommended for shallower fissures with a U or V-type profile and fissures with an extremely narrow gap. In their study, Hosoya et al found no statistical significancet differences in sealant penetration between the no-vibration and vibration groups.^12^ However, the authors concluded that sealant penetration was more affected by the shape, depth, and additional fissures than by using a vibrating probe.^12^ Our study also found that the fissure profile can affect the sealant’s penetration depth more than vibration. Kim et al studied the vibratory method of sealant application.^16^ They found that the sonic vibration groups showed increased penetration compared to the control and sealant heating groups.^16^ The penetration they found was almost 100% of the entire depth of the fissure. Such results in our study were found only in the U-profile of the fissure. In the remaining fissure profiles, penetration ranges between 48 and 86%. This is probably due to the variation on vibration frequencies used – 140 Hz in our study versus 380 Hz in the research by Kim et al.^16^

Data from our study showed that both thermal sealant groups showed greater depth penetration than the conventional group. The only exception is the IK-profile of the fissure, where there was no statistically significant difference. The viscosity of the materials varies with temperature as increasing temperature decreases viscosity and affects fluidity.^5^ Authors found that the heat group showed less penetration than the sonic vibration group.^16^ This does not coincide with the results from the current study. The tested resin sealant showed statistically significant penetration in all fissure profiles when heated to 51.0°C. Applying heat decreases the viscosity and increases the resin sealants’ penetration into the fissure. Furthermore, the extent to which vibrations can penetrate is restricted by reducing vibrational energy as the distance from the source increases.

Several studies have evaluated sealant depth penetration using different dyes.^8,19^ They have demonstrated that flowable nanocomposites show a greater fissure penetration depth than classical sealants,^12^ but few studies have focused on the essential fissure type.^21,24^ The penetration depth is influenced by both the tooth’s preparation prior to the sealant’s placement and the depth of the fissure system of the tooth itself.^19^ Clinicians often select the appropriate material for preventive sealing of the occlusal surface of teeth based on its qualities, such as depth penetration, good sealing ability, marginal adaptation, and minimal microleakage.^15^ Based on the results from the current study, we can conclude that the qualities of the material, as well as the method of its application and placement, play a criticalt role in the depth of penetration of the sealant into pits and fissure systems.

Enameloplasty allows the sealant to penetrate the fissures more efficiently and in greater depth.^19^ This method is not a good clinical practice, as proper preparation of the material – by pre-heating or using a vibrating tool during the sealant application – can achieve the same effect without disturbing the enamel surface. However, some limitations of this study should be mentioned, the most important one being that it was conducted on permanent third molars and that primary teeth were not included. Using pressurised bicarbonate powder to clean the fissures could drive some particles deep into the system, potentially reducing the effectiveness of sealant penetration. The penetration assessment was conducted using two-dimensional images, but a three-dimensional analysis would offer a clearer and more accurate comprehension of the issue.

## CONCLUSION

Penetration capabilities of sealant are greatly affected by the fissure types, the sealant type, and the method by which the sealant is applied – heated or with a vibrating tool. Heated sealants and the usage of a vibrating tool showed deeper penetration depth.  U- and V-type fissures exhibited better penetration capability than others.  For IK-fissures, the best material is a resin-based sealant, heated to 51.0°C, and I-shaped fissures have a lower penetration rate due to their narrow entrance. Low penetration is recorded when applying GIC as sealants.

### Ethics Approval and Consent to Participate

All patients gave written informed consent for using their teeth for research purposes, and all molars were irreversibly anonymised immediately after extraction. Approval from the Ethics Committee of the Medical University of Sofia was also obtained.

### Funding

This study was supported by the European Union – Next Generation EU, through the National Recovery and Resilience Plan of the Republic of Bulgaria, project No: BG-RRP-2.004-0004-C01.
